# The Interaction of Bovine β-Lactoglobulin with Caffeic Acid: From Binding Mechanisms to Functional Complexes

**DOI:** 10.3390/biom10081096

**Published:** 2020-07-23

**Authors:** Nicoleta Stănciuc, Gabriela Râpeanu, Gabriela Elena Bahrim, Iuliana Aprodu

**Affiliations:** Faculty of Food Science and Engineering, Dunarea de Jos University of Galati, 111 Domnească Street, 800201 Galați, Romania; Nicoleta.Stanciuc@ugal.ro (N.S.); grapeanu@ugal.ro (G.R.); Gabriela.Bahrim@ugal.ro (G.E.B.)

**Keywords:** caffeic acid, β-lactoglobulin, complexation, antioxidant, antidiabetic, binding, in silico

## Abstract

In this study, the interaction of native and transglutaminase (Tgase) cross-linked β-lactoglobulin (β-LG) with caffeic acid (CA) was examined, aiming to obtain functional composites. Knowledge on the binding affinity and interaction mechanism was provided by performing fluorescence spectroscopy measurements, after heating the native and cross-linked protein at temperatures ranging from 25 to 95 °C. Regardless of the protein aggregation state, a static quenching mechanism of intrinsic fluorescence of β-LG by CA was established. The decrease of the Stern–Volmer constants with the temperature increase indicating the facile dissociation of the weakly bound complexes. The thermodynamic analysis suggested the existence of multiple contact types, such as Van der Waals’ force and hydrogen bonds, between β-LG and CA. Further molecular docking tests indicated the existence of various CA binding sites on the β-LG surface heat-treated at different temperatures. Anyway, regardless of the simulated temperature, the CA-β-LG assemblies appeared to be unstable. Compared to native protein, the CA-β-LG and CA-β-LG_Tgase_ complexes (ratio 1:1) exhibited significantly higher antioxidant activity and inhibitory effects on α-glucosidase, α-amylase, and pancreatic lipase, enzymes associated with metabolic syndrome. These findings might help the knowledge-based development of novel food ingredients with valuable biological properties.

## 1. Introduction

Caffeic acid (CA) is a polyphenol produced through the secondary metabolism of vegetables [1], including olives, coffee beans, fruits, potatoes, carrots, and propolis, and constitutes the main hydroxycinnamic acid found in the diet of humans. The biological activity of CA was demonstrated in vitro and in vivo, as providing innumerable physiological effects, such as antibacterial activity [1], antiviral activity [2], antioxidant activity [3], anti-inflammatory activity [4], anti-atherosclerotic activity [5], immunostimulatory activity [6], antidiabetic activity [3], cardioprotective activity [7], antiproliferative activity [8], hepatoprotective activity [9], anticancer activity [2,3], and anti-hepatocellular carcinoma activity [10]. Additionally, Pessato et al. [11] showed that the complexation of whey protein isolate (WPI) with CA was able to attenuate oral sensitization in C3H/HeJ mice against WPI.

β-lactoglobulin (β-LG) is a small globular protein belonging to the lipocalin family, consisting of 162 amino acid residues [12]. β-LG can bind and transport many amphiphilic and hydrophobic ligands, such as phenolic compounds, fatty acids, vitamins, flavor compounds, etc. The main site of β-LG involved in the binding of ligands is the internal cavity, calyx, of the β-barrel. Also, Trp^19^, Tyr^20^, Tyr^42^, Gln^44^, Gln^59^, Gln^68^, Leu^156^, Glu^157^, Glu^158^, and His^161^ have been reported as belonging to a second binding site, while Tyr^102^, Leu^104^, and Asp^129^ to a third site [13]. It has been reported that β-LG exhibits five pH-induced transitions between pH 1 and pH 13 [14]. For example, at low pH values (around 2.00–3.00), β-LG assumes a monomeric structure due to the considerable electrostatic repulsion between the two subunits of the dimer that prevails at physiological pH levels [15]. The so-called N-to-Q transition of β-LG occurring between pH 4.5 and pH 6 is accompanied by increases in volume, whereas the Tanford transition is centered at pH 7.5 and is accompanied by volume decreases, increases in protein hydration, and loosening of the interior packing of β-LG as reflected by the 12% increase of its intrinsic compressibility [14]. The accessibility of the ligands to the β-LG calyx is pH-dependent and mediated by the mobile EF loop (residues 84 to 90)—the loop is closed when Glu^89^ is protonated, and it opens upon deprotonation [16]. All solved structures having ligands bound to the calyx exhibit an open EF loop, suggesting that the site is accessible at pH values close to neutral [17].

β-LG exhibits antibacterial properties affecting the human immune system by stabilizing cell proliferation [18]. It thus contributes to the protection against diseases such as sepsis and septic shock with subsequent multi-organ failure, which are currently increasingly frequent causes of death [19]. However, due to its structural particularities, β-LG is considered to be one of the main milk allergens, which is responsible for the allergenic effects of milk, especially among young children. However, many studies carried out on conformational β-LG changes contribute to changes in the emerging allergenicity [20]. It has been demonstrated that the binding of different ligands to β-LG changes their biological activity [11,21].

Known as a protein cross-linker, microbial transglutaminase (Tgase), is able to catalyze the reaction between the γ-carboxamide group of glutamate (Glu) and ε-amino of lysine (Lys) in proteins, thus generating covalent cross-linking through the formation of intra- and intermolecular ε-(γ-glutamyl)-lysine cross-links [22]. The enzyme is widely used to induce protein polymerization with the purpose of improving the gel strength of the resulting products. Hypothesizing the possibility of developing protein aggregates with good ability to incorporate ligands, the Tgase-assisted reaction was used in the present study to cross-link the β-LG.

We previously demonstrated that β-LG is able to bind carotenoids from the sea buckthorn extract, anthocyanins from different sources, polyphenols, etc. [23,24]. Therefore, in this study, the binding mechanism between CA and β-LG in its native and Tgase-assisted cross-linked state (n-β-LG and Tgase-β-LG) was detailed by fluorescence spectroscopy and molecular modeling for the proper understanding of the functional biologically active compound-protein interactions, and, therefore, for estimating the potential therapeutic and technological applications. Further, we hypothesized that the complexation of CA by β-LG in two molecular states, native and cross-linked, would lead to several complexes, in which the β-LG-CA complexes might present superior biological function, such as antioxidant and inhibitory effects on some enzymes involved in metabolic syndrome. Hereto, our study attempts to link different information related to structural particularities and binding mechanisms between the two studied components with enhanced functionality of the resulted complexes. To the best of our knowledge, this is the first time that the complexation of β-LG, in different molecular states with CA is studied, aiming to obtain functional composites with enhanced antioxidant, inhibitory effects towards enzymes associated with metabolic syndrome, including α-glucosidase and α-amylase, and pancreatic lipase.

## 2. Materials and Methods 

### 2.1. Materials

β-LG (purity ≥ 90%, genetic variants A and B, lyophilized powder) from bovine milk and CA (CAS 331-39-5; 95% purity) were purchased from Sigma (Sigma–Aldrich Co., St. Louis, MO). Transglutaminase (Tgase) from Activa^TM^ (Ajinomoto Corporation Inc., Tokyo, Japan), was used without further purification. All the reagents were of analytical grade. To obtain the protein solution, β-LG was weighed and dissolved in 50 mM Tris-HCl buffer solution (pH 8.0) at a protein concentration of 5 mg/mL. The protein concentration in the solution was determined by the Bradford method, as explained by Kruger [25], using bovine serum albumin at a concentration of 1 mg/mL in distilled water as a stock solution for the calibration curve. The CA stock solution was prepared by dissolving the compound in deionized water at a concentration of 5 mg/mL.

### 2.2. β-Lactoglobulin Cross-Linking

Prior to fluorescence spectroscopy and complexation studies, β-LG solutions were cross-linked using Tgase at an enzyme:substrate ratio of 1:15 (*w*/*w*), for 2 h at 25 °C. After 2 h of reaction, the enzyme was inactivated by heating at 95 °C for 5 min, in agreement with the suggestions of the producer. 

### 2.3. Heat Treatment

Plastic tubes (1 cm diameter) were filled with 0.2 mL of n-β-LG and Tgase-β-LG solutions. The samples were heated at different temperatures ranging from 25 to 95 °C for 10 min using a thermostatic water bath (Digibath-2 BAD 4, Raypa Trade, Barcelona, Spain). As previously tested by our group, the holding time chosen for the heat treatment of the protein solutions was long enough to ensure eventually irreversible structure rearrangements of the protein. The solutions were then cooled by introducing the tubes in ice water, such as to avoid further thermal denaturation.

### 2.4. Quenching Experiments

All fluorescence spectroscopy measurements were performed at 23 ± 1 °C on a LS-55 luminescence spectrometer (Perkin Elmer Life Sciences, Shelton, CT, USA) equipped with the software Perkin Elmer FL Winlab. For the quenching experiments with CA, the excitation wavelength was set at 295 nm, while the emission spectra were collected from 310 nm to 420 nm, with increments of 0.5 nm. Both the excitation and emission slit widths were set at 10 nm. Prior to fluorescent measurements, a volume of 0.15 mL of thermally (un)-treated n-β-LG and Tgase-β-LG solutions were suspended in 3.0 mL 0.5 mM Tris buffer, pH 8.0 and titrated by successive addition of CA. The Stern–Volmer constants, binding constants, number of binding sites, and thermodynamic parameters were calculated as previously reported [26]. In the case of all quenching experiments involving enzyme-assisted cross-linked samples, the Tgase-related spectra, registered under the same experimental conditions, were subtracted from the spectra of the complexes.

### 2.5. Molecular Modeling Investigations

The molecular model of β-LG (4DQ3 from the RCSB Protein Data Bank) [27] was prepared for the molecular dynamics steps through refinement and optimization in a vacuum and after solvation using explicit water molecules. A Berendsen thermostat was then simulated for heating the solvated molecule at 25 °C, 75 °C, 85 °C, and 95 °C in agreement with the laboratory scale experiment. The protein models heated at different temperatures were afterwards equilibrated, such as to reduce any temperature and energy oscillations of the systems. The molecular dynamics simulations were run in parallel conditions on an Intel^®^ Core™ 2 CPU 6300 1.86 GHz processor-based machine, using the GROMACS 4.6.1 package [28] and gromos43a1 force field to define the topology.

The β-LG models equilibrated at different temperatures were further used as receptors for CA in the molecular docking procedure. The PatchDock algorithm [29], relying on the molecules shape complementarity principles, was used for finding the best fits between β-LG equilibrated at different temperatures and CA. For each simulated temperature, the best docking results were established considering the complexes characterized by the lowest binding energy, which gives an indication of the good affinity between the two compounds of the molecular assembly. Details on the structure and interaction particularities of the molecules within the complexes were finally gathered performed using the PDBePISA [30] and Visual Molecular Dynamics software [31].

### 2.6. β-LG-Caffeic Acid Complexation

The method described by Pessato et al. [11] was used for the complexation process. After mixing the proteins solutions with the CA stock solution in proportions to achieve the molar complexation ratios of 1:1 and 1:2 (protein concentration fixed at 5 mg/mL), the complexation was carried out at 25 °C for 60 min in the dark, using 0.1 M HCl to adjust the pH to 3.5. Further, the samples were freeze-dried (CHRIST Alpha 1-4 LD plus, Osterode am Harz Germany) at −42 °C under a pressure of 10 Pa for 48 h. Afterward, the powders were collected and packed in metalized bags and kept at −20 °C until further analysis. The powders obtained through freeze-drying the samples containing the complexes formed by CA with the native protein and the cross-linked protein solutions were coded as CA-β-LG and CA-β-LG_Tgase_, respectively. The CA-β-LG and CA-β-LG_Tgase_ samples were further characterized by assessing the antioxidant activity and the ability to inhibit the α-glucosidase, α-amylase, and lipase activity.

### 2.7. Antioxidant Activity

The antioxidant activity of the CA-β-LG and CA-β-LG_Tgase_ samples was evaluated by determining the DPPH free radical-scavenging activity. The DPPH radical-scavenging effect involved the addition of 0.1 mL of sample solutions (10 mg/mL) to 3.9 mL of DPPH solution (0.1 M). After 90 min of incubation period at ambient temperature in the dark, the absorbance at 515 nm was measured. The scavenging percentage of DPPH was expressed as mMol Trolox/g DW using a calibration curve, as described by Kandi and Charles [32].

### 2.8. α-Glucosidase Inhibition

For assessing the α-glucosidase inhibitory effect of the CA-β-LG and CA-β-LG_Tgase_ samples, 50 μL of sample solutions (10 mg/mL in 0.1 M sodium phosphate buffer, PBS at pH 6.9) were added to 50 μL of 1 mg/mL α-glucosidase solution (0.1 M PBS pH 6.9), and incubated for 10 min at 25 °C. A 50 μL aliquot of 25 mM p-nitrophenyl-α-D-glucopyranoside solution in 0.1 M PBS (pH 6.9) was added and incubated at 37 °C for 25 min, followed by reading the absorbance at 405 nm. The enzyme inhibition was calculated using the equation 1 [33]:

(1) $$\%Inhibition=\frac{\left(A_{0}-A_{s}\right)}{A_{0}}$$

where *A_0_* is the absorbance of the control (blank, without sample addition) and *As* is the absorbance in the presence of the sample solutions. 

### 2.9. α-Amylase Inhibition 

For the α-amylase inhibitory effect of the CA-β-LG and CA-β-LG_Tgase_ samples, 500 μL of sample solutions (10 mg/mL in 0.1 M PBS, pH 6.9) were added to 500 μL of 1 mg/mL α-amylase solution and incubated at 25 °C for 10 min. Further, 500 μL of 1% soluble starch solution (previously dissolved in PBS and boiled for 15 min) was added and incubated for another 10 min. Finally, one mL of dinitro salicylic acid reagent was added, and the tubes were placed in a 100 °C water bath for 5 min. The mixture was diluted with 10 mL of distilled water, and the absorbance was read at 520 nm. The enzyme inhibition was calculated using equation 1 [33]. 

### 2.10. Lipase Inhibition

The method described by Costamagna et al. [34] was used to assess the lipase inhibitory effect of the CA-β-LG and CA-β-LG_Tgase_ samples. First, lipase solution (1.0 mg/mL) was mixed with the sample solutions (10 mg/mL) (ratio 1:1, *v/v*) followed by a pre-incubation step on ice for 5 min. The reaction started by adding 1 mL of sodium phosphate buffer 0.1 M (pH 7.0) supplemented with 0.6% (w/v) Triton X-100 and 0.15% (w/v) arabic gum, and 50 μL of 10 mM *p*-nitrophenyl palmitate and incubated at 37 °C for 20 min. The enzyme inhibition was estimated by using equation 1.

### 2.11. Statistical Analysis 

The experiments were performed in triplicate, and results are presented as mean values ± standard deviation. Data were analyzed with Minitab 18 software, using the one-way analysis of variance (ANOVA) and Tukey’s test for a statistical significance of *p* < 0.05.

## 3. Results and Discussion

### 3.1. Binding Mechanisms

**Figure 1:**
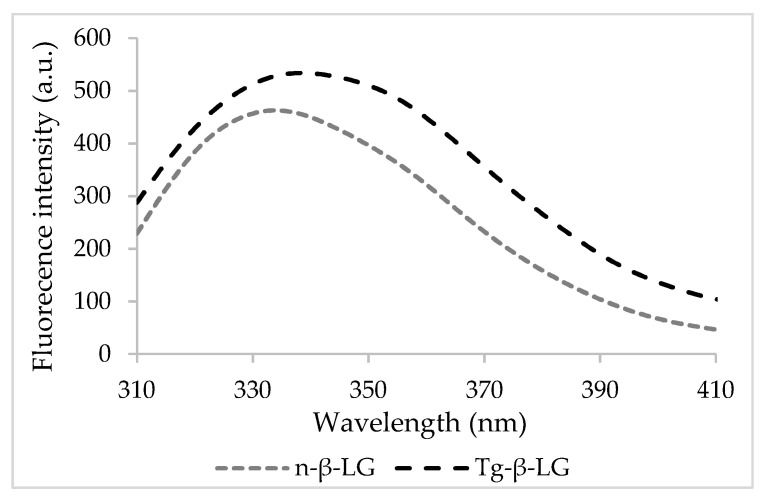
The fluorescence intensity spectra of the native and cross-linked β-lactoglobulin.

In its native state, β-LG exhibited maximum emission at a wavelength (*λ_max_*) of 334 nm, whereas when cross-linked, a significant 5 nm red-shift was found (Figure 1). The changes in the fluorescence properties of β-LG upon cross-linking suggest that the exposure and fluorescence of the Trp residues are affected by important molecular events mediated by Tgase. 

**Figure 2:**
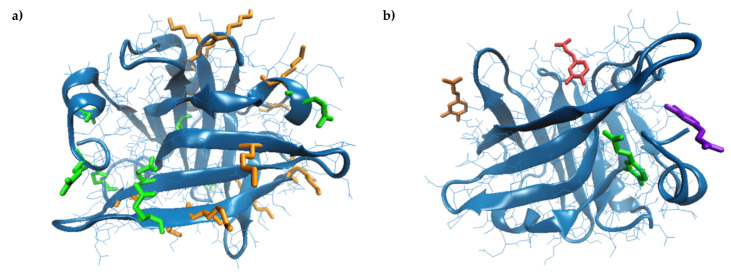
Three-dimensional model of the bovine β-lactoglobulin (4DQ3.pdb) [27] represented in a New Cartoon style. (**a**) Lys and Gln are represented in Licorice in orange and green, respectively. (**b**) Details on caffeic acid (CA) binding by β-lactoglobulin (β-LG) equilibrated at 25 °C—green, 75 °C—blue, 85 °C—ed and 95 °C—orange. The images are prepared using the Visual Molecular Dynamics software [31].

Detailed analysis of the β-LG model equilibrated at 25 °C indicated that the monomer has nine Gln and fifteen Lys residues. Out of the Gln residues, five (Gln located in positions 5, 13, 68, 115, and 159) are widely exposed to the solvent (Figure 2a) having the surface available to the solvent (SAS) in the 82.34–153.33 Å^2^ range, and only Gln^120^ appears to be completely unavailable (SAS of 0.44 Å^2^) for participating in the cross-linking reaction. Regarding Lys, all residues found in the structure of the β-LG molecule are exposed to the protein surface (Figure 2a), being available to be recognized by Tgase. Lys residues located in positions 8, 14, 69, 70, 77, 100, 138 and 141 have SAS ranging from 102.43 Å^2^ to 193.29 Å^2^, and the less exposed residues are Lys^60^, Lys^83^, and Lys^135^, with a SAS of 34.83 Å^2^, 43.70 Å^2^, and 47.48 Å^2^, respectively. Due to the large availability of the Lys and Gln residues to form *ε*-(γ-glutamyl)-lysine bonds when incubating the β-LG with Tgase [35], the probability of protein cross-linking is high, leading to high molecular weight polymers. As a consequence, the exposure and fluorescence properties of the Trp residues are affected.

**Figure 3:**
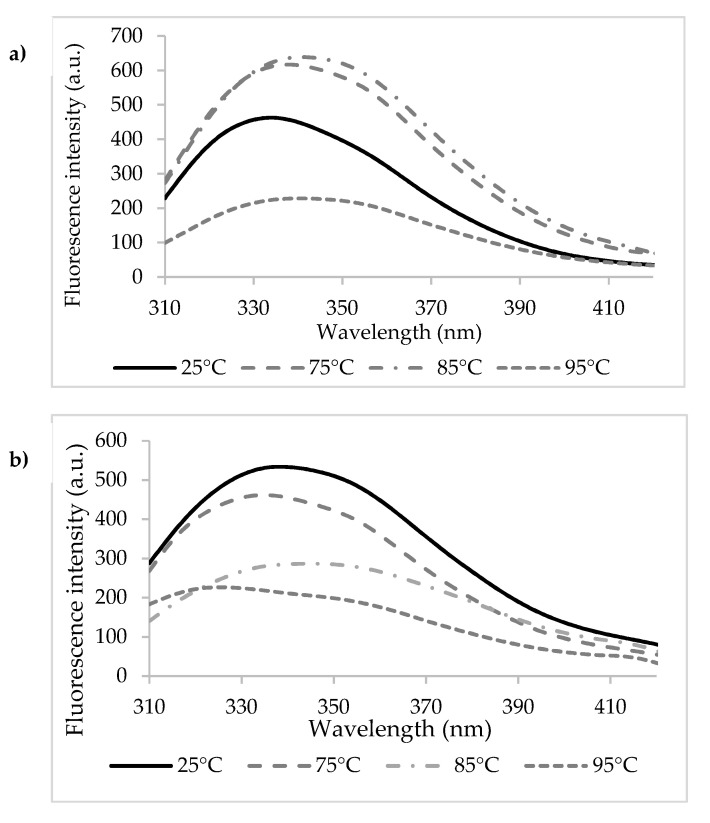
The fluorescence intensity spectra of the heat-treated β-lactoglobulin (**a**) and cross-linked β-lactoglobulin (**b**).

Heating the n-β-LG solutions caused significant redshifts of *λ_max_* from 334 nm at 25 °C to 339 nm at 75 °C, 342 nm at 85 °C, and 341 nm at 95 °C (Figure 3a). The 5–7 nm redshifts of *λ_max_* suggest that heating led to modifications in the polarity of the environment around Trp residues present in the β-LG chains. In particular, Trp^19^ became more exposed to the hydrophilic solvent as a result of heating. In agreement with Loveday [36], the temperature increase might have caused important conformational changes of the protein, potentially resulting in monomeric β-LG state and in the exposure of a hydrophobic patch and of the free thiol group of the Cys^121^. Advanced thermal treatment led to a second change in the monomer conformation, followed by the aggregation via covalent bonds mediated by Cys^121^ or non-covalent bonds (such as hydrophobic interactions). In addition to the thermal treatment, the protein concentration also influenced the mechanisms of denaturation and aggregation of whey proteins [37]. Iametti et al. [38] suggested that for the denaturation reactions in dilute protein solutions (concentration < 0.25%), the protein unfolding step was independent of the protein concentration, whereas the aggregation step was accelerated with increasing protein concentrations. 

It is well known that the intrinsic fluorescence of β-LG is mainly given by Trp^19^ residue. The Trp^19^ with emissions close to 340 nm, is buried inside β-LG at the bottom of the calyx and contributes to 80% of the total fluorescence. On the other hand, Trp^61^ (emission region 350 nm) is partly exposed to solvents and has a minor contribution to Trp fluorescence [39]. The fluorescence of Trp^61^ might be totally quenched due to the location near the disulfide bond (Cys^66^-Cys^160^) or near the guanidine group of Arg^124^. Moreover, the self-quenching by Trp^61^ of another monomer should be considered. Therefore, based on *λ_max_* values, it can be appreciated that the fluorescence intensity of β-LG at 25 °C and 75 °C is given mainly by Trp^19^, whereas by increasing the temperature up to 85 °C and 95 °C, both residues might become responsible for intrinsic fluorescence. These experimental results were validated by the *in-silico* observations. A careful analysis of Trp residues exposure was performed at the single-molecule level on the protein models equilibrated at different temperatures. Heating the protein at temperatures over 85 °C caused some structural changes involving the slight movement of Trp^19^ toward the protein surface (SAS of 4.15 Å^2^), which might have resulted in small changes in the fluorescence emission of this residue. On the other hand, the gradual burial of Trp^61^ was noticed when increasing the temperatures from 25 °C (SAS of 42.01 Å^2^) to 95 °C (SAS of 42.01 Å^2^). 

A sequential unfolding-folding process was observed when heating β-LG_Tgase_ solutions, accompanied by blue and redshifts. For example, a 3 nm blueshift was found when heating at 75 °C, followed by unfolding with a 10 nm redshift at 85 °C, and again blueshifts of 10 nm at 95 °C (Figure 3b). Therefore, sequential phenomena of exposure and blocking of Trp residues were observed, specific to unfolding processes or cross-linked network relaxation, followed by folding. Taking into account that Trp^61^ is located close to two potential participants to the Tgase-assisted cross-link reactions (Gln^59^ and Lys^60^), there is a high chance for it to be trapped within the cross-linked protein network.

In order to predict the mechanism of CA binding by β-LG, each protein solution was quenched with an increasing concentration of CA, up to 1.38 mM. The addition of CA to the n-β-LG and β-LG_Tgase_ treated at 25 °C led to significant redshifts of *λ_max_* of 5 nm and 12 nm, respectively. When thermal treatment in the temperature range of 75 °C to 95 °C was applied, significant redshifts between 10–13 nm in the case of n-β-LG and 12–25 nm in the case of β-LG_Tgase_ were found. These results suggested that interactions between n-β-LG and CA led to significant changes in the polarity of the environment around Trp residues, with exposure to the hydrophilic solvent as a result of the interaction with CA [40].

When quenching with CA, the *K_SV_* values significantly decreased (*p* < 0.05) up to 95 °C (Table 1), suggesting that the accessibility of CA decreased with increasing temperatures, in the case of both investigated molecular states. Due to the decrease in the Stern–Volmer values as a function of temperature, the mechanism of binding was assessed as static. This meant that due to the increase of the temperature, the dissociation of weakly bound complexes increased, and consequently, the Stern–Volmer constants decreased [41]. Ligands have different affinities for proteins, as suggested by various studies. For example, Gholami and Bordbar [42] reported binding of naringenin to bovine β-LG *K_SV_* values of 0.13 × 10^6^ M^-1^ at 25 °C, whereas Arroyo-Maya et al. [43] suggested a decrease in *K_SV_* values at lower temperatures (25–45 °C). 

The estimated binding constant (*K_b_*) and the number of binding sites (*n*) are shown in Table 1. The binding constants were found to increase in the tested temperature range, suggesting an increase in the binding affinity. At each tested temperature, the *n* value was lower than 1, suggesting the presence of one binding site for CA in β-LG molecules in its different states. However, the *n* values increased with increasing temperatures up to 95 °C, from 0.56 ± 0.02 to 0.66 ± 0.10 in n-β-LG and from 0.65 ± 0.07 to 1.32 ± 0.28 in β-LG_Tgase_, indicating the increase of β-LG affinity for CA. It may be concluded that the cross-linked β-LG showed greater affinity towards CA, which increased with increasing temperatures.

The thermodynamic parameters provided information necessary to understand the molecular forces that drove the complex formation, and were estimated by plotting the natural logarithm of *K_a_* versus *T^-1^.* Ross and Subramanian [44] quantified the sign and magnitude of the thermodynamic parameters related to various individual types of interactions that may have taken place in the protein-ligand binding, such as: (i) *ΔH* > 0 and *ΔS* > 0, hydrophobic force; (ii) *ΔH* < 0 and *ΔS* < 0, Van der Waals’ force and hydrogen bonding; (iii) *ΔH* < 0 and *ΔS* > 0, electrostatic interactions. Therefore, considering the thermodynamic characteristics summarized in Table 2, the negative *ΔH* and *ΔS* values suggest that Van der Waals’ force and hydrogen bonding play a major role in the β-LG and CA binding interaction, regardless of the molecular state. 

### 3.2. In Silico Investigation on Caffeic Acid Binding by β-LG 

The molecular modeling approach was further employed to find out the heat-induced changes that essentially influence the β-LG ability to bind CA. The temperature increase caused the gradual reduction of the protein surface exposed to the solvent, suggesting the overall folding on a different pattern compared to the native protein. Anyway, the reduction of the free energy of protein folding (ΔG^f^) from −151.9 kcal/mol at 25 °C to −166.0 kcal/mol at 95 °C was registered, indicating that β-LG had high thermal stability. Important molecular rearrangements were observed, accompanied by the dynamics of the hydrophobic patches’ exposure at increasing temperatures. Gradual burial of Cys residues involved in the disulfide bridges, which stabilize the hydrophobic core of β-LG occurred at temperatures over 75 °C, resulting in the slight length increase of both Cys^66^–Cys^160^ and Cys^106^–Cy^119^, which is in good agreement with Stănciuc et al. [45]. Important C-S-S-C angles torsion were registered, affecting the secondary structure motifs involving these residues, which caused the slight expansion of the cavity between the CD loop and the strands G and H [46]. Moreover, because of the thermal-induced oscillations resulting in changes in the distance and the relative position between different amino acids, the salt bridges participating in the specific protein folding varied with temperature. In particular, at temperatures over 75 °C, a new salt bridge between Asp^137^–Lys^141^ appeared to play a central role in stabilizing the protein surface. On the other hand, the temperature increase caused a disruption of the salt bridges established between Asp^53^–Lys^75^, Glu^74^–Lys^83^, and Asp^85^–Lys^83^ in the protein equilibrated at 25 °C. As a consequence, a wider opening of a small cavity involving these amino acids was observed at 95 °C on the protein surface, which appeared to bind with the highest affinity to the CA molecule. In fact, the CA binding site varied with prior thermal treatment applied to the protein (Figure 2b). As a consequence, the interaction surface and energy varied with the temperature (Table 3). In all the studied cases, the ligand binding occurred at the protein surface (Figure 2b) to small cavities with an area ranging from 239.55 Å² to 417.47 Å² and a depth ranging from 4.88 Å to 11.69 Å (Table 3). Our results comply with Harvey et al. [47], who suggested that, in addition to the central cavity, known as *calyx*, β-LG is able to bind different hydrophobic molecules to the clefts located on the surface.

It is generally accepted that the forces driving the specific interaction of proteins with their ligands depend on the energy exchanged between all molecules present in the systems, namely the protein, ligand, water, and possible ions. The analysis of single-molecule levels indicated that, regardless of the simulated temperature, the leading role in stabilizing the β-LG-CA complex was played by the hydrophobic interactions and hydrogen bonds (Table 3). The free energy of binding (ΔG^int^) included the changes of the solvation energy as well as the electrostatic and contact-dependent interactions, such as the hydrogen bonds between atoms of the two components of the complexes. The effect of the hydrogen bonds across the interface accounted for −0.44 kcal/mol. In all the studied cases, the rigid-body entropy changed at dissociation, TΔS^diss^ of 2.8 kcal/mol outweighed the free energy, −ΔG^int^, suggesting that the system moved toward a dissociated state [48]. Moreover, the negative values of ΔG^diss^ (Table 3) indicated that the dissociation of the complexes might have occurred spontaneously. These observations are in agreement with the fluorescence spectroscopy results regarding the Stern–Volmer constants decreases with the temperature increase.

### 3.3. Effect of Complexation on Antioxidant Activity

The antioxidant activity of the CA-β-LG and CA-β-LG_Tgase_ samples was compared with the antioxidant activity of the native protein before complexation. β-LG showed antioxidant activity of 0.52 ± 0.06 mMol Trolox/g DW, whereas the 1:1 complexation led to a significant increase of up to 15.02 ± 0.40 mMol Trolox/g DW for CA-β-LG and 23.86 ± 0.76 mMol Trolox/g DW for CA-β-LG_Tgase_. When using a complexation ratio of 1:2, the CA binding to the protein molecules led to the increase in antioxidant activity up to 32.24 ± 1.96 mMol Trolox/g DW and 45.26 ± 2.69 mMol Trolox/g DW in case of the CA-β-LG and CA-β-LG_Tgase_ sample, respectively. The higher antioxidant activity of the CA-β-LG_Tgase_ samples may have been due to the cross-linked network mediated by the enzyme, favoring the entrapment of a higher amount of CA.

### 3.4. Impact of Complexation on Enzyme Inhibitory Effect

In 2016, more than 1.9 billion adults worldwide (18 years and older), were overweight, and over 650 million were obese. In addition, 41 million children under the age of five were overweight or obese [49], and it is projected to be the seventh leading cause of death in 2030, with type 2 diabetes (T2DM) representing more than 90% of all diabetes cases, associated mainly with genetics and lifestyle features. Kwon et al. [50] suggested that the main strategy to reduce the breaking down of starch into sugar monomers, thus lowering the postprandial blood glucose levels, was to find proper therapeutic approaches for the control of T2DM in terms of inhibitors of carbohydrate-hydrolyzing enzymes, such as α-amylase and α-glucosidase. Additionally, one of the most promising approaches to reduce the energy intake through gastrointestinal mechanisms was the inhibition of lipases, especially pancreatic lipase [51].

In this study, three enzymes involved in carbohydrate and fat metabolism were selected to estimate the potential therapeutic strategy to suppress the production and absorption of glucose from the gastrointestinal tract. The inhibitory effect of β-LG towards α-glucosidase was 57.13 ± 1.31%, whereas the samples with complexation ratios of 1:1 and 1:2 displayed significantly lower inhibitory activity (Table 4).

The α-amylase inhibitory activity of β-LG (25.63 ± 0.80%) was significantly lower compared to the CA-β-LG and CA-β-LG_Tgase_ samples at ratios of 1:1 (Table 4). On the other hand, the inhibition exerted by the CA-β-LG and CA-β-LG_Tgase_ samples with a complexation ratio of 1:2 were at least three times lower (Table 4), suggesting that these samples are less effective in interfering with the activity of the α-amylase.

The main prescribed treatment for weight management and obesity consist of the inhibition of pancreatic lipase, involved in the splitting of triacylglycerols into absorbable monoacylglycerol and fatty acids. In our study, the inhibitory effect of the CA-β-LG and CA-β-LG_Tgase_ samples towards pancreatic lipase was lower than the values reported for different polyphenols from tea, soybean, mate tea, peanut, or grapes [52]. β-LG showed an inhibitory effect of 23.58 ± 2.14%, whereas values of 8.74 ± 0.93% and 14.23 ± 2.31% were found for CA-β-LG and CA-β-LG_Tgase_ samples with a complexation ratio of 1:1, respectively (Table 4). Increasing the molar ratio in the complex to 1:2 led to a significant decrease (*p* < 0.05) in the lipase inhibitory effect.

Our results suggest that the complexation of β-LG with CA may lead to ingredients, with enhanced antioxidant activity, which might be able to reduce glucose uptake/absorption. The obtained powders showed good inhibition capacity towards both enzymes associated with glucose metabolism, with higher efficiency for the samples with a complexation ratio of 1:1. However, Costamagna et al. [33] suggested that the simultaneous inhibition of both enzymes would result in abnormal bacterial fermentation in the colon, due to the presence of undigested carbohydrates.

Based on the above-mentioned results, it could be assumed that the increased concentration of the caffeic acid in the complexes led to an increase of antioxidant activity and lowered the inhibitory effect towards α-amylase, α-glucosidase, and pancreatic lipase. In addition, enzymatic cross-linking led to a significant increase in the antioxidant capacity of the complexes and enhanced the inhibitory effect towards the enzymes associated with metabolic syndrome.

## 4. Conclusions

This work investigated the binding affinity, interaction mechanism, and structural aspects of the interaction between caffeic acid and β-lactoglobulin in native and cross-linked states, based on fluorescence spectroscopy and modeling methods. The analysis of the fluorescence quenching data revealed that caffeic acid quenched the intrinsic fluorescence of β-lactoglobulin and had a stronger binding affinity towards cross-linked protein. The quenching mechanism was found to be static for both molecular states of the protein. The negative *ΔH* and *ΔS* values suggested that Van der Waals’ force and hydrogen bonding played the major role in the β-LG and CA binding interaction, regardless of the molecular state. The in-silico approach was used to check the amino acids involved in the interaction with CA. Due to the thermally induced structural changes, different cavities located on the β-LG surface pre-treated at different temperatures ranging from 25 °C to 95 °C were found to accommodate the ligand. Accordingly, the interaction surface and binding energy varied with the thermal treatment applied to the proteins. Regardless of the simulated temperature, the analysis performed at the single-molecule level indicated that the β-LG—CA assemblies are unstable.

The complexes formed by the native and cross-linked proteins with CA in different molar ratios led to powders with significant antioxidant activity and were active against enzymes associated with metabolic syndrome such as α-glucosidase, α-amylase, and pancreatic lipase. The cross-linked powders obtained in a molar ratio of 1:1 showed a higher inhibitory effect towards α-amylase, α-glucosidase, and pancreatic lipase. These findings are valuable for the scientific community and food producers willing to develop novel food ingredients and foods for the treatment/prevention of life-threatening diseases.

## Figures and Tables

**Table 1:** Binding parameters between β-LG in native and cross-linked states and caffeic acid at different temperatures.

	n-β-LG	β-LG_Tgase_	n-β-LG	β-LG_Tgage_	n-β-LG	β-LG_Tgase_
T (°C)	*K_SV_* (10^6^ Mol^-1^)		*K_b_* (10^6^ Mol^-1^)		*n*	
25	25.46 ± 0.72 ^a1^	35.33 ± 1.41 ^a^	0.72 ± 0.03 ^c^	0.52 ± 0.03 ^b^	0.56 ± 0.02 ^a^	0.65 ± 0.07 ^b^
75	21.89 ± 0.42 ^b^	27.50 ± 1.53 ^b^	0.83 ± 0.10 ^b^	0.77 ± 0.06 ^a,b^	0.63 ± 0.06 ^a^	0.81 ± 0.08 ^b^
85	20.24 ± 0.10 ^c^	14.50 ± 0.70 ^c^	0.89 ± 0.01 ^a,b^	0.85 ± 0.03 ^a,b^	0.69 ± 0.01 ^a^	0.89 ± 0.04 ^b^
95	17.34 ± 0.54 ^d^	12.50 ± 1.12 ^c^	0.96 ± 0.01 ^a^	1.04 ± 0.21 ^a^	0.66 ± 0.10 ^a^	1.32 ± 0.28 ^a^

^1^ Means within a column that do not share a superscript letter are significantly different at *p* < 0.05.

**Table 2:** Thermodynamic parameters for binding between β-LG in native and cross-linked states and caffeic acid at different temperatures.

	T(K)	ΔH (J/Mol)	ΔS (J/Mol·K)	ΔG (J/Mol)
n-β-LG	298	−418.72 ± 11.58	−14.87 ± 1.36	4012.54 ± 21.34
	348			4756.04 ± 14.32
	358			4904.74 ± 12.21
	368			5053.44 ± 14.58
Tgase-β-LG	298	−983.76 ± 23.45	−16.42 ± 2.01	3915.95 ± 16.78
	348			4738.05 ± 19.09
	358			4902.47 ± 18.74
	368			5066.89 ± 14.53

The thermodynamic values indicate that the binding of CA and β-LG is mainly entropy-driven, and the enthalpy is unfavorable for it.

**Table 3:** Single molecule level details on the interaction between β-lactoglobulin (β-LG) equilibrated at different temperatures and caffeic acid (CA).

Descriptors	Temperature, °C			
	25	75	85	95
Total protein surface, Å^2^	8546.19 ± 88.69	8585.87 ± 170.85	8212.59 ± 130.76	8167.92 ± 125.47
Hydrophobic protein surface, Å^2^	4953.24 ± 67.28	4760.91 ± 134.34	4597.87 ± 120.93	4605.74 ± 94.34
βLG –CA interface area, Å^2^	216.2	210.9	178.0	129.3
*Particularities of the binding site*				
Amino acids interacting with CA	Tyr^20^, Tyr^42^, Val^43^, Glu^44^, Gln^59^, Cys^66^, Gln^68^, Leu^156^, Glu^157^, Glu^158^, Gln^159^, Cys^160^, His^161^	Gln^35^, Arg^40^, Tyr^42^, Trp^61^, Cys^66^, Glu^158^, Gln^159^, Cys^160^, His^161^, Ile^162^	Leu^31^, Pro^38^, Leu^39^, Val^41^, Leu^58^, Lys^60^, Asn^90^, Met^107^, Asn^109^, Gln^115^, Ser^116^	Glu^74^, Lys^75^, Thr^76^, Lys^83^, Ala^86^, Leu^87^,
Amino acids involved in HB with CA (HB length)	Leu^156^ (2.1Å)	Gln^35^ (3.51Å), Gln^159^ (2.92Å)	Lys^60^ (2.79Å)	Thr^76^ (3.01Å), Lys^83^ (3.24Å)
Total cavity surface, Å²	293.69	417.47	239.55	402.18
Total cavity depth, Å	9.42	9.37	4.88	11.69
βLG–CA binding energy, kcal/mol	−23.23	−24.75	−18.33	−19.72
ΔG^f^, kcal/mol	−151.9	−158.6	−158.3	−166.0
ΔG^int^, kcal/mol	2.6	1.9	1.2	0.8
TΔS^diss^, kcal/mol	2.8	2.8	2.8	2.8
ΔG^diss^, kcal/mol	−5.4	−4.7	−4.0	−4.6

ΔG^f^—free energy of folding, ΔG^int^—free energy of binding, ΔG^diss^—free energy of complex dissociation, TΔS^diss^—rigid-body entropy changes at dissociation.

**Table 4:** Inhibitory effect of complexes between caffeic acid (CA) and β-LG on enzymes involved in carbohydrates and fats metabolism.

Enzymes	Native β-LG	Complexation Ratio 1:1		Complexation Ratio 1:2	
		Native β-LG-CA	Cross-linked β-LG-CA	Native β-LG-CA	Cross-linked β-LG-CA
*Enzymes related to carbohydrate metabolism*					
α-Glucosidase	57.13 ± 1.31 ^a1^	47.12 ± 2.60 ^b^	44.95 ± 4.28 ^b^	35.36 ± 3.52 ^c^	36.22 ± 3.38 ^c^
α-Amylase	25.63 ± 0.80 ^b^	44.27 ± 0.33 ^a^	40.38 ± 3.62 ^a^	11.89 ± 0.24 ^c^	15.02 ± 1.21 ^c^
*Enzymes related to fat metabolism*					
Lipase	23.58 ± 2.14 ^a^	8.74 ± 0.93 ^c^	14.23 ± 2.31 ^b^	2.21 ± 0.24 ^d^	4.42 ± 0.53 ^d^

^1^ Means within a line that do not share a superscript letter are significantly different at *p* < 0.05.
